# Microglial Immunometabolism in Alzheimer’s Disease

**DOI:** 10.3389/fncel.2020.563446

**Published:** 2020-09-18

**Authors:** Daniel C. Shippy, Tyler K. Ulland

**Affiliations:** Department of Pathology and Laboratory Medicine, University of Wisconsin, Madison, WI, United States

**Keywords:** Alzheimer’s disease, microglia, immunometabolism, neuroinflammation, neurodegeneration, glucose, ketone bodies

## Abstract

Alzheimer’s disease (AD) is a neurodegenerative disorder characterized by amyloid-β (Aβ) plaques and the formation of neurofibrillary tangles (NFTs) composed of hyperphosphorylated tau. In response to Aβ and tau aggregates, microglia, the primary innate immune cells of the central nervous system (CNS), facilitate Aβ and tau clearance and contribute to neuroinflammation that damages neurons. Microglia also perform a wide range of other functions, e.g., synaptic pruning, within the CNS that require a large amount of energy. Glucose appears to be the primary energy source, but microglia can utilize several other substrates for energy production including other sugars and ketone bodies. Recent studies have demonstrated that changes in the metabolic profiles of immune cells, including macrophages, are important in controlling their activation and effector functions. Additional studies have focused on the role of metabolism in neuron and astrocyte function while until recently microglia metabolism has been considerably less well understood. Considering many neurological disorders, such as neurodegeneration associated with AD, are associated with chronic inflammation and alterations in brain energy metabolism, it is hypothesized that microglial metabolism plays a significant role in the inflammatory responses of microglia during neurodegeneration. Here, we review the role of microglial immunometabolism in AD.

## Introduction

Alzheimer’s disease (AD) is an age-related neurodegenerative disorder associated with memory loss and impaired cognitive abilities. AD is a major cause of disability and dependency in the United States and worldwide, causing a significant impact on not only the individual patient, but also their family, community, and the healthcare system (Collaborators, 2019). To date, no effective treatment for AD exists, so advances in our understanding of AD neuropathology, and the associated immune responses, are necessary to develop therapeutic strategies to combat AD.

AD neuropathology is a complex process with several key features. Macroscopically, the AD brain displays cortical atrophy mostly affecting the medial temporal lobes (Serrano-Pozo et al., 2011). On the cellular level, AD is characterized by the accumulation of extracellular amyloid-β (Aβ) plaques followed by the formation of intracellular neurofibrillary tangles (NFTs) composed of hyperphosphorylated tau (p-tau) resulting in synapse loss (Holtzman et al., 2011). In response to the accumulation of Aβ plaques and NFTs, microglia are activated and facilitate Aβ and tau clearance in addition to inducing a neuroinflammatory response that damages neurons suggesting a delicate balance between a beneficial, detrimental, or mixed microglia reaction to AD progression (Lue et al., 2010; Leyns and Holtzman, 2017).

Early studies of immunometabolic functions focused on the requirements of certain metabolites to provide energy and support biosynthesis in activated macrophages (Oren et al., 1963; Newsholme et al., 1986; Fukuzumi et al., 1996). Numerous recent studies suggest changes in intracellular metabolic pathways in immune cells can alter their functions (Chang et al., 2013; Huang et al., 2014; O’Neill et al., 2016; Zhao et al., 2020). The role of metabolism in neuron and astrocyte function (Pfrieger and Ungerer, 2011; Turner and Adamson, 2011; Jha and Morrison, 2018), has been studied while until recently the role of cellular metabolism in microglia has been less well understood. Considering many neurological disorders are associated with inflammation and alterations in energy metabolism in the brain, it is hypothesized that microglial metabolism plays a significant role in the inflammatory responses of microglia during neurodegeneration associated with AD. Here, we review the role of microglial immunometabolism in AD. We discuss our understanding of the overall role of microglia in AD, metabolism in the brain, and the importance of glucose and ketone body metabolism in AD.

## Microglia and AD

Microglia, the innate immune cells of the central nervous system (CNS), account for 10–15% of the adult glial cell population in the brain (Nayak et al., 2014). Microglia develop in the yolk sac and migrate to the developing CNS during embryogenesis where they can continuously self-renew without support from bone marrow-derived precursor cells (Ginhoux and Prinz, 2015). Microglial activation in AD was first described over 100 years ago by Alois Alzheimer (English Translation: Alzheimer et al., 1995). Recently, significant progress has been made in our understanding of how microglia develop, function, and participate in AD (Lue et al., 2010; Ulrich et al., 2014, 2017; Condello et al., 2015; Vincenti et al., 2015; Wang Y. et al., 2015; Wang et al., 2016; Ulrich and Holtzman, 2016; Yuan et al., 2016; Keren-Shaul et al., 2017; Ulland et al., 2017; Gotzl et al., 2019; Mathys et al., 2019; Schlepckow et al., 2020; Zhou et al., 2020).

**Figure 1:**
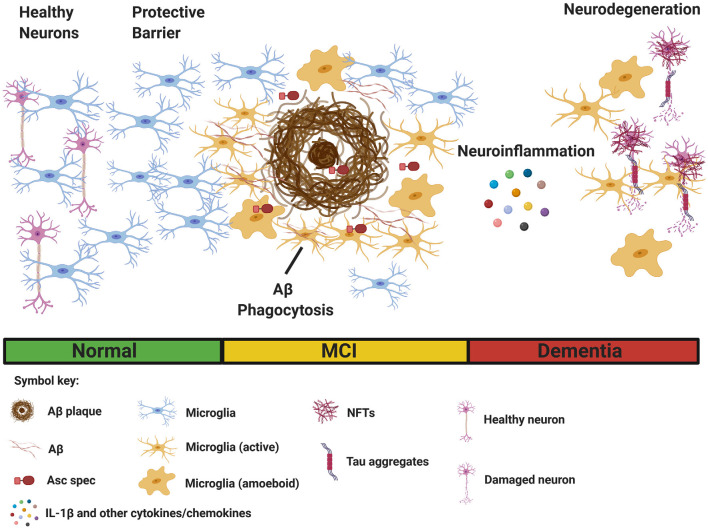
Multiple functions of microglia in Alzheimer’s disease (AD). Microglia sense pathological Aβ accumulation in the brain, and rapidly respond to the site of injury. (Left) Initially, microglia provide protective functions by facilitating Aβ clearance through phagocytosis to restore tissue homeostasis. Microglia also acts as a protective barrier to inhibit plaque expansion and contact with adjacent, healthy neurons. (Right) In contrast, sustained microglial activation promotes detrimental effects such as inflammasome activation and the secretion of IL-1β and other inflammatory cytokines and chemokines, leading to neuroinflammation. Asc specs, formed following NLRP3 inflammasome activation, and released by microglia, also seed new Aβ plaques. Finally, chronic neuroinflammation enhances the aggregation of hyperphosphorylated tau in neurofibrillary tangles (NFTs), resulting in neurodegeneration. (Bottom) Clinical stages of AD [normal, mild cognitive impairment (MCI), and dementia]. The figure was created with BioRender.com.

Despite a great deal of progress, the precise role of microglia in AD is not completely understood. Several reports suggest microglial activation in the early stages of AD delays disease progression through clearance of soluble and oligomeric Aβ (Frackowiak et al., 1992; Qiu et al., 1997; Frautschy et al., 1998). Activated microglia are hypothesized to reduce Aβ accumulation through phagocytosis mediated clearance (Qiu et al., 1997, 1998; Frautschy et al., 1998; Figure 1). Electron microscopy data show that microglia rapidly respond to Aβ deposition, extending their processes, and engulfing Aβ (Frackowiak et al., 1992). Additionally, microglia can act as a barrier to decrease the neurotoxic effects of plaque contact on adjacent neurons (Condello et al., 2015; Wang et al., 2016; Yuan et al., 2016; Figure 1). Overall, there is an abundance of data to suggest that proper microglia function protects against pathology early in AD development, however, in contrast to these findings, plentiful evidence also exists that microglia can be neurotoxic and contribute to neurodegeneration in AD. Microglia are directly linked to synapse loss (Wu et al., 2015; Spangenberg and Green, 2017) and provoke tauopathy-mediated pathology (Leyns and Holtzman, 2017; Leyns et al., 2017). Furthermore, evidence suggests tau pathology itself can stimulate microglial activation (Morales et al., 2013).

Microglia are hypothesized to exhibit functional plasticity within the context of neurodegenerative diseases (Jha et al., 2016). Many factors, such as complement, can influence microglial polarization (Bohlson et al., 2014). Activated microglia can somewhat resemble classically activated macrophages, formerly referred to as M1 macrophages, release pro-inflammatory cytokines (IL-1β, IL-18, TNF-α, IFN-γ, and IL-6), produce reactive oxygen species, and other pro-inflammatory molecules implicated in neurodegeneration in AD (Nayak et al., 2014; Wang W. Y. et al., 2015; Spangenberg and Green, 2017). In contrast, microglia may assume a phenotype similar to alternatively activated macrophages resulting in enhanced phagocytosis and anti-inflammatory responses (Park et al., 2016; Figure 1). The M1/M2 phenotype hypothesis, remains very controversial, as distinct microglial polarization has not been properly supported by research findings (Ransohoff, 2016).

Regardless of whether microglia provide a protective, pathogenic, or mixed contribution, in AD it is clear microglia is a key player in AD progression. Therefore, understanding characteristics of microglia during AD progression, like metabolism, may lead to novel approaches to treat and/or prevent AD.

## Metabolism in AD

While the brain only makes up about 2% of the total human body mass it accounts for approximately 25% of the glucose and 20% of the oxygen consumed by the body (Attwell and Laughlin, 2001; Alle et al., 2009). The underlying reasons for the high energy demand of the brain include neurotransmitter reuptake, action potential generation, and the generation and renewal of ion gradients (Attwell and Laughlin, 2001). The brain is therefore highly sensitive to changes in energy supply, with minor alterations in energy processing linked to hindered brain function and neurodegenerative disorders, like AD (Edison et al., 2013; Andersen et al., 2017; Skotte et al., 2018).

A growing body of evidence suggests AD pathology is driven by metabolic dysfunction (de la Monte and Tong, 2014). Diabetes appears to be an important risk factor for developing AD (Baumgart et al., 2015) with several studies linking diabetes and impaired insulin signaling in the brain to AD pathogenesis (Biessels et al., 2006; Bomfim et al., 2012; Talbot et al., 2012; Zhao et al., 2017; Kim et al., 2019). Overall, diabetics are at a greater risk for AD and the brains of individuals with AD have higher levels of insulin, insulin receptor, and insulin signaling (Hoyer, 2004; Steen et al., 2005; Craft et al., 2013). These studies highlight the importance of brain energy metabolism in AD development and provide a solid basis for the hypothesis that microglial immunometabolism is a critical component of the inflammatory responses of microglia in AD development.

## Microglial Immunometabolism of Glucose in AD

Glucose is the main energy source for microglia, and they express several glucose transporters (Maher et al., 1994; Duelli and Kuschinsky, 2001), with GLUT1 (SLC2A1) and GLUT3 (SLC2A3) being the major isoforms (Kalsbeek et al., 2016; Wang et al., 2019). A study of genes associated with energy metabolism in mouse microglia, astrocytes, and neurons indicates microglia express the required genes for both glycolytic and oxidative energy metabolism (Zhang et al., 2014). A large scale proteomic study also identified several proteins expressed by activated microglia linked to sugar metabolism, highlighting the importance of microglial sugar metabolism in AD (Johnson et al., 2020). Additional studies show non-activated microglia depend on oxidative phosphorylation for ATP production while activated microglia rely on glycolysis (Bernhart et al., 2010). Further studies validated these findings showing that LPS induced significant metabolic changes in BV2 cells resulting in decreased mitochondrial function and increased glycolysis (Voloboueva et al., 2013; Gimeno-Bayon et al., 2014). Additionally, microglial response to LPS is enhanced under high glucose conditions as indicated by significantly increased release of IL-6 and TNF-α (Zhang et al., 2015).

Of further interest are the number of recent studies which suggest the nod-like receptor family pyrin domain containing 3 (NLRP3) inflammasome is activated by glycolytic enzymes (Hughes and O’Neill, 2018); activation of the NLRP3 inflammasome has been demonstrated to contribute to AD pathology (Heneka et al., 2013; Venegas et al., 2017; Ising et al., 2019). For example, inhibition of the mammalian target of rapamycin complex 1 (mTORC1) suppressed hexokinase 1-dependent glycolysis and caspase-1 activation, implicating NLRP3 inflammasome activation in macrophage metabolism (Moon et al., 2015). A further study showed hyperglycolysis and hexokinase induction activates microglia and is essential for neuroinflammation by microglia under hypoxic conditions (Li et al., 2018). Furthermore, microglia that have activated the NLRP3 inflammasome switch their metabolism towards glycolysis which has the potential to impact energy-requiring processes, like phagocytosis (Rubio-Araiz et al., 2018). Interestingly, the addition of anti-TLR2 increased microglial phagocytosis of Aβ with decreased expression of an important glycolysis enzyme, 6-phosphofructo-2-kinase/fructose-2,6-biphosphatase. These findings were indirectly linked to the inhibition of inflammasome activation by the anti-TLR2 antibody (Rubio-Araiz et al., 2018).

Recently, a link between defective microglial function associated with triggering receptor on myeloid cells (TREM2) and glucose metabolism in neurodegeneration has been identified (Kleinberger et al., 2017; Ulland et al., 2017). Mutations in *TREM2* are associated with an increased risk for the development of AD (Guerreiro et al., 2013; Jonsson et al., 2013; Song et al., 2017). *TREM2* T66M knock-in mice displayed an age-dependent decline in microglial activity along with a significant decrease in cerebral blood flow and brain glucose metabolism suggesting a potential microglial function in managing brain glucose metabolism (Kleinberger et al., 2017). TREM2 was also found to play a major role in microglial metabolic fitness (Ulland et al., 2017). *Trem2*^−/−^ 5XFAD mice were less metabolically competent as they exhibited large decreases in glycolytic and mammalian target of rapamycin (mTOR) activity compared to wild-type cells; decreases in mTOR signaling were associated with increased autophagy. Additionally, the metabolic deficiency, and lack of microglial responsiveness, was restored in *Trem2*^−/−^ 5XFAD mice by increasing microglial energy capacity with cyclocreatine (Ulland et al., 2017). Together, these studies highlight the importance of microglial metabolism of glucose in AD. Further investigation is needed to determine the precise mechanisms in which microglial metabolism of glucose influences AD pathology.

## Microglial Immunometabolism of Ketone Bodies in AD

Microglia can use ketone bodies as an alternative energy source to glucose. The three main ketone body components are acetate, β-hydroxybutyrate (BHB), and acetoacetate (Laffel, 1999). Levels of ketone bodies increase during periods of extended exercise, starvation, caloric restriction, or in individuals on low carbohydrate diets, e.g., the ketogenic diet. Dietary regimens, like a ketogenic diet, have been shown to reduce inflammation and suppress microglial activation; therefore, there is interest in using the ketogenic diet as a potential therapeutic option for AD. Ketones are known to have a protective effect in AD by improving synaptic plasticity and reducing oxidative stress (Yin et al., 2016). BHB activates G-protein-coupled receptor 109A (GPR109A), also called hydroxycarboxylic acid receptor 2 (HCA2), which attenuates NF-κB signaling, pro-inflammatory enzyme (Cox-2 and iNOS), and cytokine (IL-6, TNF-α, and IL-1β) production in both macrophages and microglia (Rahman et al., 2014; Fu et al., 2015; Huang et al., 2018). Although it appears ketone body metabolism by microglia has a significant role in AD, much work is needed to elucidate the mechanistic insights into how this metabolism modulates microglial activity and function.

## Targeting Microglial Immunometabolism for Therapeutic Use

To date AD drug discovery research has focused on tauopathy or Aβ reduction. As discussed above, glycolysis is a major factor in maintaining activated microglia, while non-activated microglia rely more on oxidative metabolism (Bernhart et al., 2010). Based on this data, it is reasonable to suggest that reprogramming microglia towards oxidative metabolism may be a useful therapeutic strategy to reduce neuroinflammation in AD. The study by Gu et al. (2017) shows a reduced expression of Clock (clk)1, a mitochondrial hydroxylase, enhanced inflammation, and aerobic glycolysis in microglia by an NF-κB-dependent mechanism. Additionally, their study showed that inhibition of glycolytic metabolism abolished the enhanced inflammatory phenotype seen in Clk1-deficient BV2 cells (Gu et al., 2017). Based on this observation, several molecules could be potential therapeutic candidates, including dimethyl fumarate and its metabolite, monomethyl fumarate, as they have been shown to inhibit NF-κB activity (Gillard et al., 2015; Al-Jaderi and Maghazachi, 2016; Kornberg et al., 2018). Also, short-chain fatty acids, like dichloroacetate and butyrate, could potentially be used as therapeutics, as both have been shown to promote metabolic shifts away from glycolysis towards oxidative metabolic pathways (Blouin et al., 2011; Matt et al., 2018).

Microglia shift to an anti-inflammatory phenotype in response to BHB (Huang et al., 2018). Additionally, studies in macrophages (Youm et al., 2015) and primary microglia (Deora et al., 2017) indicate BHB blocks NLRP3 inflammasome activation. In macrophages, BHB can block NLRP3 inflammasome activation by preventing potassium efflux, which in turn reduces apoptosis-associated speck-like protein containing a caspase recruitment domain (ASC) oligomerization and ASC speck formation (Youm et al., 2015). Whether this mechanism is similar in microglia remains unclear. These findings suggest BHB treatment or dietary regimens that promote elevated BHB levels, could be a promising therapy for AD. Indeed, several studies of mice on a ketogenic diet have shown reduced tau and amyloid pathologies (Van der Auwera et al., 2005; Kashiwaya et al., 2013). BHB also protects against AD pathology by targeting multiple aspects of AD pathogenesis (Wu et al., 2020). BHB administration to 5XFAD mice improved cognitive functions, decreased microgliosis, and reduced Aβ accumulation (Wu et al., 2020). Furthermore, several studies in humans demonstrate a ketogenic diet may improve cognitive abilities in patients with neurodegenerative disorders, with higher ketone levels correlating with improved cognitive functioning (Reger et al., 2004; Henderson et al., 2009; Krikorian et al., 2012; Taylor et al., 2018; Ota et al., 2019). In contrast, five-month-old mice on a ketogenic diet for three months did not improve cognition in the amyloid or tau mouse model of AD (Brownlow et al., 2013). This study, however, showed improvement in the motor performance of mice, suggesting BHB may enhance existing neuron function without modifying the rate of neuropathy in AD (Brownlow et al., 2013). Additionally, BHB did not inhibit synuclein fibril mediated inflammasome activation in microglia, suggesting that activation of NLRP3 by synuclein fibrils acts through different mechanisms compared to adenosine triphosphate (ATP) and monosodium urate (MSU) activation (Deora et al., 2017). Based on these contrasting findings, further work is needed to determine if BHB is a viable treatment option for AD.

Medium-chain triglyceride diets have been developed to provide a more palatable alternative to the ketogenic diet (Huttenlocher et al., 1971). Caprylic triglyceride (CT) is a medium-chain triglyceride, which is metabolized into ketone bodies that can be used as an alternative energy source for neuronal metabolism (Bach and Babayan, 1982) and has been developed as a medical food therapy to promote mitochondrial metabolism in AD (Roman, 2010). Several studies suggest an increase in brain ketone metabolism can increase overall brain energy supply to improve mild cognitive impairment (MCI; Croteau et al., 2018; Fortier et al., 2019; Neth et al., 2020). Apolipoprotein E (ApoE) appears to be an important factor in the efficacy of this therapy, as carriers of the *APOE4* allele do not see the improvement in cognitive function as subjects administered CT who are not carriers of the *APOE4* allele (Reger et al., 2004; Henderson et al., 2009; Farah, 2014; Yamazaki et al., 2019). While CT administration is generally thought to function through the generation of ketones to provide an alternative energy source for brain cells, including microglia, the underlying mechanisms, however, are still largely unknown. Further characterization of all forms of the ketogenic diet might improve and increase their use as a therapeutic regime for AD.

Another approach to AD treatment is to target microglial genes important in microglial metabolism. As previously discussed, TREM2 is vital to microglial metabolic fitness (Ulland et al., 2017). Therapeutic strategies that promote TREM2 expression and function may have beneficial effects in AD patients (Ulland and Colonna, 2018). For example, TREM2 signaling could potentially be increased by using small molecule inhibitors or agonistic antibodies to phospholipid ligands, and inhibiting protease-mediated cleavage could increase TREM2 expression. Furthermore, since TREM2 maintains microglial mTOR metabolism and signaling, the use of metabolic agents that promote microglial metabolic fitness may also be a viable option (Ulland and Colonna, 2018). However, the use of these potential therapies is questionable as there is conflicting evidence about the impact of modifying TREM2 signaling in a tau model of AD. Leyns et al. suggest microglial TREM2 signaling is detrimental during disease progression, as *TREM2* deficiency results in decreased neuroinflammation and protects against neurodegeneration (Leyns et al., 2017). In contrast, the study by Bemiller et al. demonstrated that deficiency of microglial *TREM2* increases tau pathology (Bemiller et al., 2017). Further investigation is needed, as much is to be learned before therapeutic agents targeting TREM2 signaling, microglia, and metabolism in AD can be developed and implemented clinically.

## Future Perspectives

The emerging field of immunometabolism has provided significant progress in our understanding of how cellular and systemic metabolism affects immune responses. More importantly, these data suggest that targeting immune cell metabolism may be a valuable strategy for the development of advanced therapeutics to treat human disease (Bettencourt and Powell, 2017; Matsushita and Pearce, 2018). Little is known, however, about microglial immunometabolism in the context of neurodegeneration and AD. A major challenge in targeting microglia-specific metabolism as a therapeutic strategy is to determine the possible conflicting functions microglia may have in AD progression. As discussed above, there is evidence to suggest microglia may have a beneficial and/or detrimental effect during AD pathogenesis depending on several factors, including the stage of AD progression. Further work is necessary to address these concerns and design microglia-targeted therapeutic strategies for AD intervention.

## Author Contributions

DS drafted the manuscript. TU reviewed and edited the manuscript. All authors contributed to the article and approved the submitted version.

## Conflict of Interest

The authors declare that the research was conducted in the absence of any commercial or financial relationships that could be construed as a potential conflict of interest.
